# Comparative Transcriptome Reveals ART1‐Dependent Regulatory Pathways for Fe Toxicity Response in Rice Roots

**DOI:** 10.1111/ppl.70398

**Published:** 2025-07-14

**Authors:** Yoshiaki Ueda, Naoki Yamaji, Matthias Wissuwa

**Affiliations:** ^1^ Crop, Livestock and Environment Division Japan International Research Center for Agricultural Sciences Tsukuba Ibaraki Japan; ^2^ Institute of Plant Science and Resources Okayama University Kurashiki Okayama Japan; ^3^ PhenoRob Cluster and Institute of Crop Science and Resource Conservation (INRES) University of Bonn Bonn Germany

**Keywords:** ART1, gene co‐expression analysis, iron toxicity, reactive oxygen species, rice

## Abstract

Iron (Fe) is an essential element for plants, but an excess supply can have detrimental effects. Fe toxicity induces complex physiological and genetic responses, and due to this complexity, the knowledge of transcriptional regulatory mechanisms under Fe toxicity is very limited. Previous studies suggested that plant responses to excess Fe involve oxidative stress caused by reactive oxygen species (ROS), which itself causes transcriptional changes. We hypothesized that dissecting these complex responses could lead to the identification of a novel factor and conducted a comparative transcriptome analysis using roots of rice plants exposed to nutrient solutions containing 1 or 5 mM of hydrogen peroxide (a major form of ROS) or 300 mg L^−1^ of Fe (as FeSO_4_). Genes induced by hydrogen peroxide overlapped with 62%, 49%, and 30% of Fe toxicity‐upregulated genes at 3 h, 1 day, and 3 days following treatment initiation. Subsequent gene co‐expression analyses classified genes into 21 groups with varying responsiveness to ROS and Fe toxicity. Genes in group 15 were specifically upregulated by Fe toxicity and overlapped significantly with aluminum (Al)‐inducible genes and target genes of the Zn‐finger transcription factor, ART1, which regulates Al response in rice roots. Additional experiments using the *art1* knock‐out mutant demonstrated that ART1 is crucial for upregulating genes such as *STAR2* and *FRDL4* in response to Fe toxicity. This study reveals the contribution of ART1‐dependent regulatory pathways in rice roots under Fe toxicity.

## 1 Introduction

Iron (Fe) is an essential nutrient for plants, and plants need to absorb an appropriate amount of Fe for optimal growth. Fe deficiency commonly occurs when the solubility of Fe is low, such as in high‐pH conditions, calcareous soils, and drylands, and typically results in leaf chlorosis, reduced photosynthesis, and decreased grain yield. On the other hand, Fe toxicity occurs when the lower soil redox status favors the reduction of insoluble Fe^3+^ and increases the concentration of easily soluble Fe^2+^ in the soil solution. Such a highly reduced soil environment is common for lowland rice production, and Fe toxicity is a specific problem that lowland rice encounters. Fe toxicity mainly occurs in tropical rice‐growing countries in Africa and Southeast Asia (Becker and Asch 2005; van Oort 2018) and typically causes necrotic leaves and reduced photosynthesis. These negative effects result in more than 10% yield loss in rice, potentially leading to complete plant loss in severe cases (Sahrawat 2005). Therefore, it is considered a major issue in wetland rice production (Audebert and Fofana 2009; Haefele et al. 2014). To decipher tolerance mechanisms and develop resistant varieties, it is essential to understand the physiological and genetic mechanisms relevant to the response to Fe toxicity.

Contrary to Fe deficiency responses, which have been extensively studied and the related response mechanisms are well understood (Kobayashi and Nishizawa 2012; Kobayashi et al. 2014), the mechanisms of transcriptional response and tolerance to Fe toxicity are far from fully understood (Wairich, Aung, et al. 2024). While excess Fe uptake is the primary cause of Fe toxicity symptoms, Fe toxicity can also lead to mineral deficiencies and part of the responses are likely triggered by an imbalance of essential nutrients, such as potassium and magnesium, as indicated by changes in stress levels when the status of these nutrients is altered (Tanaka and Tadano 1972; Suriyagoda et al. 2017; Wu et al. 2019; Kirk et al. 2022; Rajonandraina, Ueda, et al. 2023). Apart from nutrient‐related factors, secondary responses caused by excess Fe supply may also impact plant responses. Free Fe in plants participates in the Fenton reaction and produces harmful molecules collectively known as reactive oxygen species (ROS) (Becana et al. 1998; Wu et al. 2017). Indeed, treatment with excess Fe increased ROS levels (Wu et al. 2017; Wairich, Wang, et al. 2024), which itself triggers a significant change in the transcriptome in both roots and shoots (Frei et al. 2010; Ashrafuzzaman et al. 2018; Mabuchi et al. 2018). Thus, the response of plants to Fe toxicity is considered a combination of different types of physiological and genetic responses. Accordingly, a previous time‐course transcriptome study suggested that the gene expression patterns depend on the duration of Fe toxicity treatment, highlighting the contribution of multiple factors at different time scales (Aung, Masuda, et al. 2018).

Mutant studies have identified several genes involved in the response and tolerance to Fe toxicity in rice. Knock‐out lines of a hemerythrin domain‐containing protein, HRZ2, increased Fe accumulation in shoots and susceptibility to Fe toxicity (Aung, Kobayashi, et al. 2018). A knock‐out line of a potassium transporter, AKT1, reduces potassium uptake, allowing more Fe accumulation in shoots and decreasing tolerance (Wu et al. 2019). A knock‐out line of *NAS3*, one of the genes involved in phytosiderophore biosynthesis (Lee et al. 2009), showed increased foliar Fe concentrations and resulted in attenuated tolerance to Fe toxicity (Aung et al. 2019). In addition to these nutrient‐related genes, *GSNOR* encoding an S‐nitrosoglutathione reductase confers tolerance to Fe toxicity by reducing the content of nitric oxides (Li et al. 2019). These studies highlight the importance of both nutrient‐ and oxidative stress‐related components in Fe toxicity tolerance, but much remains to be elucidated regarding the transcriptional pathways and genes involved in the responses.

Gene co‐expression analysis is a versatile systems biology tool to dissect complex regulatory patterns underlying a certain response. In this approach, genes are classified into different groups termed “modules” (Langfelder and Horvath 2007), and genes in the same module are assumed to be under a similar regulatory mode (Ma et al. 2013). Co‐expression network analysis is effective in revealing intricate transcriptional regulatory pathways and was previously used to systematically identify transcription factors that regulate secondary cell wall synthesis in rice (Hirano et al. 2013). A similar approach was utilized for dissecting the response of *Brachypodium distachyon* to combinations of salinity, drought, and heat stress, revealing a group of genes that are particularly important for combinatorial stress responses (Shaar‐Moshe et al. 2017). Thus, gene co‐expression analysis, unlike simple differential expression analysis, holds the potential to provide insights into complex gene regulatory networks. Since Fe toxicity response likely involves multiple physiological responses, its regulatory mode may be clarified by using a co‐expression analysis strategy.

Based on these, we hypothesized that Fe toxicity responses that likely involve authentic nutrient responses and oxidative stress responses can be dissected through a systems biological approach that allows us to identify novel regulatory pathways involved in Fe toxicity response. We limited the analysis to roots, which are in direct contact with high concentrations of Fe and respond more strongly to Fe toxicity than aboveground tissues (Aung, Masuda, et al. 2018). Our specific objectives were: (1) to elucidate to what extent each of oxidative stress and components other than oxidative stress explain the whole Fe toxicity responses, (2) to elucidate novel genes important for the response to Fe toxicity, and (3) to demonstrate the involvement of candidate genes by a mutant study.

## 2 Materials and Methods

### 2.1 Plant Materials and Growth Conditions

Seeds of rice (cv. Nipponbare) were surface‐sterilized with a 5% NaClO solution for 5 min, rinsed with tap water, and placed on a moistened tissue paper in a petri dish. The seeds were germinated at 28°C–30°C for 2 days, then transferred to a mesh tray floating on deionized water and covered with a lid. After 2 days, the lid was removed, and the solution was exchanged for 0.1 mM CaCl_2_ and 12 μM Fe‐EDTA. After an additional 3 days, the solution was exchanged for the Ca + Fe solution that also contained 0.05× concentration of K, Mg, and micronutrients (B, Cu, Mn, Mo and Zn). These solutions were refreshed every 2–3 days. Two weeks after germination, seedlings were transferred to 13‐L hydroponic containers containing half‐strength Yoshida solution (Yoshida et al. 1976). After 1 week, the solution was exchanged for full‐strength Yoshida solution and cultured for an additional week. Before the onset of treatment, the pH of the Yoshida solutions was adjusted to 5.5–6.0 with NaOH solution 2–3 times per week. The full‐strength Yoshida solution was composed of 1.42 mM NH_4_NO_3_, 100 μM NaH_2_PO_4_, 0.5 mM K_2_SO_4_, 1 mM CaCl_2_, 1 mM MgSO_4_, 36 μM Fe‐EDTA (equivalent to 2 mg L^−1^ Fe), 9 μM MnCl_2_, 18.5 μM H_3_BO_3_, 0.16 μM CuSO_4_, 1.5 μM ZnSO_4_, and 0.07 μM (NH_4_)_6_Mo_7_O_24_. All the experiments were conducted in a temperature‐controlled greenhouse (30°C/25°C for day/night) with natural sunlight, occasionally with an additional LED light to ensure a minimum light intensity of 300 μmol m^−2^ s^−1^.

#### 2.1.1 Experiment 1 (H_2_O_2_ Treatment for RNA‐Seq Study and Dose‐Dependent Analysis of Fe Toxicity Responses)

Roots of 24‐day‐old plants were treated with 1× Yoshida solution containing 0, 0.2, 1, or 5 mM H_2_O_2_, concentrations similar to those in a previous report (Mabuchi et al. 2018). The treatment was initiated at 14 o'clock and harvested 3 h after the treatment started. The whole root was flash‐frozen with liquid nitrogen and stored at −80°C until further analyses. In the same experiment, another set of plants was treated with a control solution (2 mg L^−1^ Fe as Fe‐EDTA) and a control solution added with 20, 80, or 300 mg L^−1^ Fe as FeSO_4_, which are within the realistic range of Fe concentrations in soil solutions that typically induce Fe toxicity (Becker and Asch 2005). For the analysis of Fe response, all solutions (including control solutions) contained 0.1% (final concentration) of melted agar (Wako, # 016‐11875) to reduce the redox status of the solution and effectively induce Fe toxicity (Wang et al. 2008). The whole root was harvested 3 h after the onset of the treatment. It was confirmed that FeSO_4_ solution corresponding to 300 mg L^−1^ Fe had barely detectable Al concentration (< 20 nM; Figure S1) that did not induce plant responses. RNA samples obtained from three biological replicates were analyzed by RNA‐seq.

#### 2.1.2 Experiment 2 (Fe Toxicity Treatment for RNA‐Seq Study)

26‐day‐old plants were treated with 1× Yoshida solution (control) or 1× Yoshida solution containing an additional 300 mg L^−1^ Fe as FeSO_4_. The solution for Fe toxicity treatment, as well as the solution for the control group, contained 0.1% melted agar, similar to Experiment 1. The treatment was initiated at 14 o'clock, and the whole root was harvested 3 h, 1 day, and 3 days after the onset of the treatment, snap‐frozen with liquid nitrogen and stored at −80°C until further analyses. RNA samples obtained from three biological replicates were analyzed by RNA‐seq.

#### 2.1.3 Experiment 3 (Phenotypic and Gene Expression Analysis of *art1* Mutant)

Twenty nine‐day‐old wild‐type Nipponbare (WT) and previously reported *art1* mutant plants (*art1‐2*; Huang et al. 2012) were treated with 1× Yoshida solution (control) or 1× Yoshida solution containing an additional 300 mg L^−1^ Fe as FeSO_4_, including 0.1% of melted agar as in Experiment 1. Root samples for gene expression analysis were harvested 3 h after the onset of the treatment with liquid nitrogen and stored at −80°C until further analyses. For phenotypic evaluation, each treatment group had two containers. The treatment lasted for 11 days.

#### 2.1.4 Experiment 4 (Gene Expression Analysis of *art1* Mutant)

Twenty nine‐day‐old WT and *art1* plants were treated with the control or Fe toxicity solution as in Experiment 3. Root samples were harvested 3 h after the treatment initiation using liquid nitrogen and stored at −80°C until the analysis.

### 2.2 Plant Phenotyping

Fe toxicity‐induced foliar symptoms were evaluated by scoring the leaf bronzing score (LBS) (Wu et al. 2014) using the two most recently fully expanded leaves with a 0–10 scale. Shoot and root were separated, dried at 70°C for > 3 days, and weighed.

### 2.3 RNA Sequencing and Data Analysis

Total RNA was extracted from frozen pulverized samples using the RNeasy Plant Mini Kit (Qiagen) for Experiments 1, 2, and 4 and the ISOSPIN Plant RNA kit (Nippon Genetics) for Experiment 3 following the manufacturer's instructions, including DNase treatment. The sequencing library was constructed using the total RNA using the Standard mRNA Library Prep kit. Sequencing was carried out using HiSeq X (Illumina) to generate 150‐bp paired‐end reads. Raw sequencing data were analyzed essentially, as reported previously (Ueda 2024). Raw fastq files were checked for quality using fastqc software (https://www.bioinformatics.babraham.ac.uk/projects/fastqc/). Regions with low quality were trimmed using trimmomatic with the following settings: LEADING:15 TRAILING:15 SLIDINGWINDOW:10:15 MINLEN:50, during which adapter sequences were also removed. The trimmed reads were mapped to the Nipponbare reference genome (IRGSP‐1.0) using hisat2 (Kim et al. 2015). After converting the resultant sam files to bam format with samtools (Li et al. 2009), expression was quantified using stringtie (Pertea et al. 2015). A multidimensional scaling (MDS) plot was created using DESeq2 (Love et al. 2014).

Differentially expressed genes were analyzed using count data of abundantly expressed genes (i.e., transcript per million [TPM] > 2 across all samples) using the nbinomLRT command in DESeq2. Adjusted *p*‐values < 0.05 were considered significant.

### 2.4 Gene Co‐Expression Analysis

The R software WGCNA (Langfelder and Horvath 2008) was used for the analysis. The count data obtained from RNA‐seq of 27 samples were processed using the vst command in DESeq2 to stabilize variation among samples. Groups of co‐expressed genes, referred to as “modules”, were identified using the blockwiseModules command in WGCNA with the expression matrix generated by the vst function with the following settings: power = 12, networkType = “signed”, minModuleSize = 30, and mergeCutHeight = 0.3. The expression of the “eigengene”, defined as the first principal component of the expression values of all genes within each module (Langfelder and Horvath 2007), was calculated for each sample group.

### 2.5 Real‐Time PCR

cDNA was synthesized from total RNA using PrimeScript RT Master Mix reagent (Takara). Gene expression was quantified by real‐time PCR using the CFX96 Real‐Time PCR Detection System (Bio‐Rad) with TB Green *Premix Ex Taq* II (Takara) and gene‐specific primers (Table S1). The expression level was determined using the standard curve method with an internal reference gene *OsC3H38* (Höller et al. 2014).

### 2.6 Statistical Analyses

Overlap of gene lists and the corresponding *p*‐values were analyzed based on Fisher's exact test using the R package GeneOverlap (https://github.com/shenlab‐sinai/GeneOverlap). Two‐way analysis of variance (ANOVA) for qPCR analysis was conducted to analyze the single effects of genotype, treatment, and their interaction using the multcomp (Hothorn et al. 2008) and car (Fox and Weisberg 2019) R packages with a significance threshold of *α* = 0.05. Phenotype data from Experiment 3 were analyzed using the lmerTest (Kuznetsova et al. 2017) and lsmeans (Lenth 2016) packages, designating treatment, genotype, and their interaction as fixed effects and containers as random effects, with a significance threshold of *α* = 0.05. Gene ontology enrichment analysis was performed on the RiceFrend website (Sato et al. 2012), and terms with corrected *p*‐values (FDR) < 0.05 and no less than three gene entries were considered significant.

## 3 Results

### 3.1 Dissection of Reactive Oxygen Species‐Responsive Genes With Fe‐Responsive Genes

**FIGURE 1:**
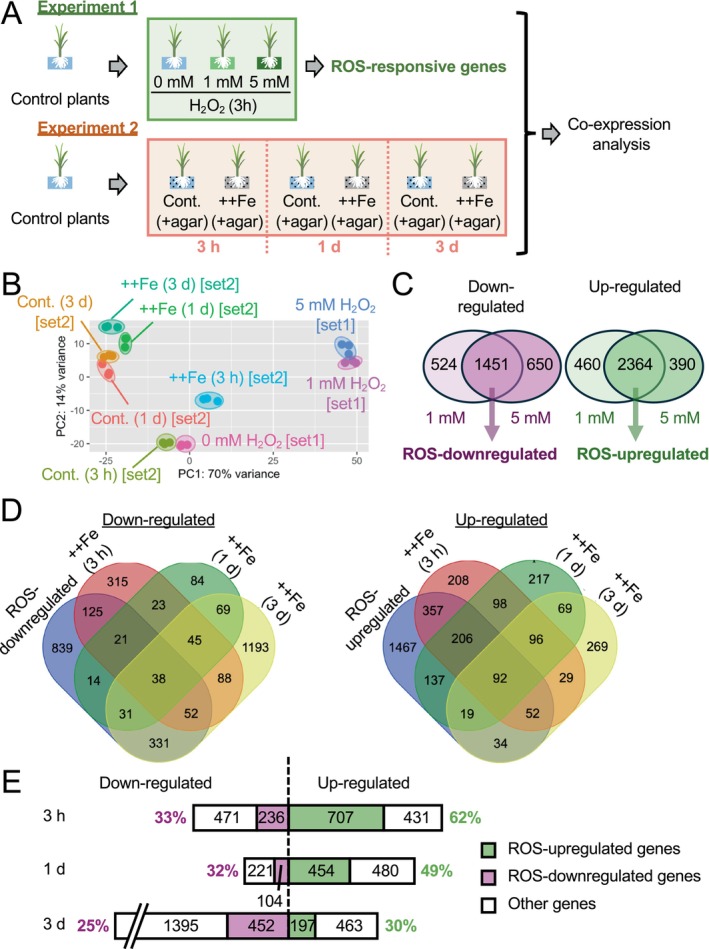
Response of rice roots to H_2_O_2_ and Fe toxicity. (A) The experimental design. “Cont.” and “++Fe” indicate the control and Fe toxicity conditions, respectively. (B) Multidimensional plot created using RNA‐sequencing data from 27 root samples representing 9 conditions mentioned in A. (C) Venn diagrams of genes responsive to 1 and 5 mM H_2_O_2_. Genes commonly affected by both 1 mM and 5 mM H_2_O_2_ in the same direction were defined as “ROS‐suppressive” or “ROS‐inducible” genes. (D) Venn diagrams showing the overlap of ROS‐responsive genes and Fe toxicity‐responsive genes at different time points. (E) Number and ratio of ROS‐responsive genes among Fe toxicity‐responsive genes at different time points. The numbers within and beside bars indicate the number of genes in each category and the ratio of ROS‐responsive genes, respectively.

Hydrogen peroxide (H_2_O_2_) is a major form of ROS with a relatively long half‐life that impacts various physiological processes, inducing gene expression (Das and Roychoudhury 2014; de Abreu Neto and Frei 2016; Mabuchi et al. 2018). To define ROS‐responsive genes, roots of rice plants were treated with H_2_O_2_ stress (3 h) and analyzed by RNA‐seq (Figure 1A). The MDS plot shows that the transcriptome patterns were strongly affected by H_2_O_2_ treatment (Figure 1B). Roots treated with 1 mM or 5 mM of H_2_O_2_ had 2824 and 2756 upregulated genes, respectively. Similarly, 1975 and 2101 genes were significantly downregulated by 1 and 5 mM H_2_O_2_ treatment (Figure 1C). Given the similar responses triggered by both H_2_O_2_ concentrations, the commonly up‐ and down‐regulated genes (2364 and 1451 genes) were defined as ROS‐upregulated and ROS‐downregulated genes (Figure 1C).

In a separate experiment, Fe toxicity‐responsive genes were identified at 3 h, 1 day, and 3 days from the onset of the treatment. The number of genes upregulated by Fe toxicity was highest at 3 h (1138), which gradually declined over time, with 934 and 660 at 1 day and 3 days, respectively. The number of genes downregulated by Fe toxicity was highest at 3 days (1847), followed by 3 h (707) and 1 day (325). The comparison of gene expression patterns in the above experiments enabled us to distinguish ROS‐responsive genes from those more specifically responding to nutrient imbalances. A total of 897 ROS‐upregulated genes overlapped with Fe toxicity‐upregulated genes at least at one time point, whereas 612 ROS‐downregulated genes overlapped with Fe toxicity‐downregulated genes at least at one time point (Figure 1D). The ratio of ROS‐responsive genes among Fe toxicity‐responsive genes was highest at 3 h, accounting for 33% and 62% of down‐ and up‐regulated genes. The ratio of ROS‐inducible genes among Fe toxicity‐upregulated genes declined over prolonged treatment (Figure 1E). Besides the ROS‐upregulated genes, the number of Fe toxicity‐upregulated genes remained almost constant over time, with 431, 480, and 463 genes for 3 h, 1 day, and 3 days of treatment. Although the number of Fe toxicity‐downregulated and ROS‐downregulated genes was highest at 3 days, the ratio of ROS‐suppressive genes among all Fe toxicity‐suppressive genes was less variable, with 33%, 32%, and 25% for 3 h, 1 day, and 3 days of treatment (Figure 1E).

### 3.2 Classification of ROS‐ and Fe Toxicity‐Responsive Genes Through Co‐Expression Analysis

**FIGURE 2:**
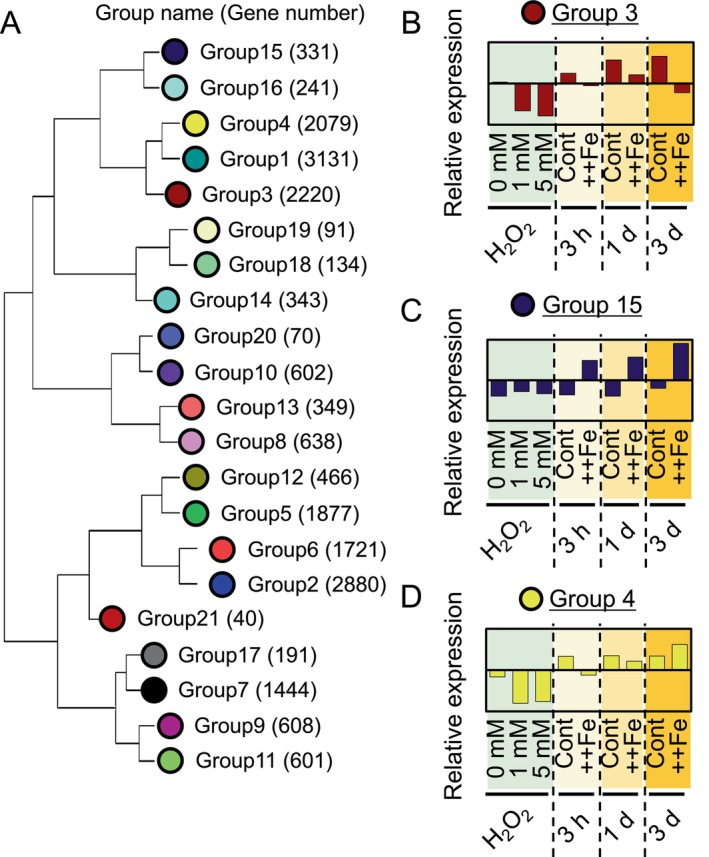
Clustering of genes through co‐expression analysis. (A) Dendrogram showing the relationships among different co‐expression groups. The numbers in parentheses indicate the gene number in each group. A specific color was assigned to each group for better visibility. (B–D) The expression of eigengene, which represents the first principal component of the expression of genes within the group, for group 3 (B), group 15 (C), and group 4 (D) under 9 different conditions. Data represent the means of 3 samples. The y‐axis scale is consistent across all graphs.

To define groups of genes that exhibit similar expression patterns under various conditions, a gene co‐expression analysis was carried out using the WGCNA software (Langfelder and Horvath 2008) and the RNA‐seq results obtained from 27 samples. A total of 20,835 abundantly expressed genes in roots were classified into 21 gene groups, with 784 genes not falling into any group (Figure 2A; Table S2). For easier recognition, a color code is hereafter assigned to each group. The “eigengene” is defined as the first principal component of genes within each group (Langfelder and Horvath 2007) and illustrates the overall expression pattern of genes in each module. The eigengene expression was highly variable among different treatments (Figures 2B–D and S2). The analysis of eigengene expression showed that some groups were enriched with genes responsive to both H_2_O_2_ and Fe toxicity. For instance, gene group 3 (brown) consisted of genes downregulated by both H_2_O_2_ and Fe toxicity, thus representing ROS‐downregulated and Fe toxicity‐downregulated group (Figure 2B). This group was enriched with gene ontology (GO) terms such as “response to oxidative stress” and “antioxidant activity”, confirming its relation to ROS response (Table S3). This module contained MYB transcription factor family genes involved in stress response (e.g., *MYB19*, *MYB55/61*), genes related to Fe utilization such as *YSL16* (Kakei et al. 2012), as well as *GSNOR* related to Fe toxicity tolerance by controlling the level of nitric oxide (Li et al. 2019). Likewise, group 7 (black), also suppressed by both H_2_O_2_ and Fe toxicity, included pathogen‐ and ROS‐ related genes such as WRKY transcription factor family genes (e.g., *WRKY13*, *WRKY39*, *WRKY107*). Group 6 (red) was induced by both H_2_O_2_ and Fe toxicity and contained antioxidant‐related genes such as *APX1* that encodes an ascorbate peroxidase (Agrawal et al. 2003) and *ERF131/SERF1* encoding a transcription factor involved in salinity and H_2_O_2_ response (Schmidt et al. 2013) (Table S2). In line with the significant overlap between ROS‐ and Fe toxicity‐responsive genes (Figure 1D,E), all ROS‐responsive modules showed responsiveness to Fe toxicity to a certain extent (Figures 2 and S2).

The expression of genes in some modules strongly responded only to Fe toxicity but not to H_2_O_2_. The eigengene for group 15 (midnightblue) was markedly induced by Fe toxicity but not by H_2_O_2_ (Figure 2C). This group included *MGT1* that encodes a Mg transporter (Chen et al. 2012), Al‐responsive genes such as *STAR1/2* (Huang et al. 2009), phosphorus deficiency‐responsive genes such as *SPX2/5* (Shi et al. 2014; Wang et al. 2014), and Fe toxicity‐inducible genes such as *FER1/2* (Stein et al. 2009) (Table S2). It is worth noting that the induction of P deficiency‐related genes could be partly due to the precipitation of P caused by excess Fe, which resulted in an available P concentration of 5 μM, in contrast to 100 μM in the original solution (Figure S3).

Some gene groups exhibited time‐dependent expression patterns. Genes in group 4 (yellow) were suppressed by ROS and Fe toxicity at 3 h (Figure 2D). However, the degree of suppression weakened after 1 day of treatment, and after 3 days, the expression pattern reversed from 3 h. Genes such as *FRDL1*, involved in root‐to‐shoot Fe translocation (Yokosho et al. 2009) and *VIT2*, which encodes a vacuolar Fe transporter (Zhang et al. 2012) were included in this group (Table S2).

### 3.3 Meta‐Analysis of Transcriptome With Previously Defined Nutrient‐Responsive Genes

**FIGURE 3:**
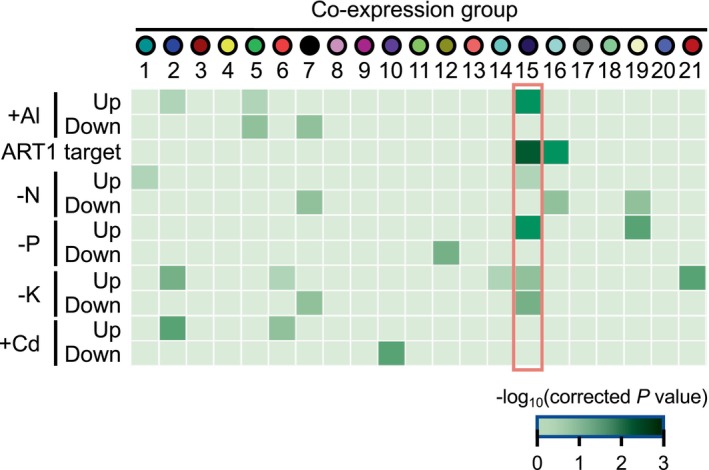
Overlap of genes within each module and nutrient‐responsive genes. The significance levels of the overlap of nutrient‐responsive genes and module genes are shown as a heatmap. *p*‐values were calculated using the GeneOverlap R package and Fisher's exact test.

To further characterize the groups, the members of each group were compared with the previously defined nutrient‐responsive genes in rice roots (Table S4). Some of the gene groups detected by the co‐expression analysis were enriched with nutrient‐responsive genes (Figure 3). For example, group 10 (purple), which is suppressed by both H_2_O_2_ and Fe toxicity, significantly overlapped with genes suppressed by cadmium (Cd) stress. Group 15 (midnightblue), which strongly responds to Fe toxicity but to a lesser extent to H_2_O_2_, exhibited a remarkable overlap with P deficiency‐inducible and Al‐inducible genes (Figure 3). The genes in group 15 did not significantly overlap with Cd‐inducible genes, suggesting that they do not represent general heavy metal stress‐responsive genes.

Al resistance transcription factor 1 (ART1) is a C2H2 Zn‐finger transcription factor that serves as a master regulator for Al response in rice (Yamaji et al. 2009; Arbelaez et al. 2017). Due to a highly significant overlap between Al‐responsive genes and genes in group 15, the overlap of ART1‐downstream genes and genes in group 15 was examined. The previously defined 31 ART1‐downstream genes (Yamaji et al. 2009) showed even stronger overlap with the genes in group 15 and, to a lesser extent, with group 16 (Figure 3).

### 3.4 Time‐ and Dose‐Dependency of ROS‐ and Fe Toxicity‐Responsive Genes

**FIGURE 4:**
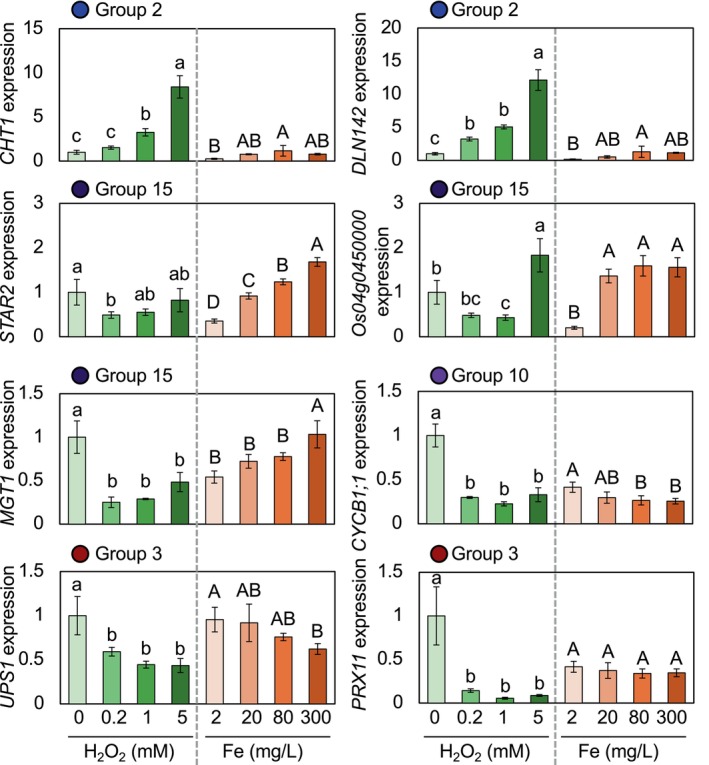
Dose‐dependent expression of selected genes under H_2_O_2_ and Fe toxicity stress. The expression of representative genes from different co‐expression groups after 3 h of H_2_O_2_ or Fe toxicity treatment is shown. Data represent mean ± standard deviation (*n* = 4). One‐way ANOVA was conducted for each treatment group (H_2_O_2_ and Fe toxicity), and significant differences are indicated by different alphabets.

The dose‐dependent expression of ROS‐ and nutrient‐responsive genes from different co‐expression groups was examined. The levels of stress treatment (0, 0.2, 1, and 5 mM H_2_O_2_ and 2, 20, 80 and 300 mg L^−1^ of Fe) were found to be optimal for inducing various strengths of genetic responses (Figure 4). In line with the eigengene expression, genes in group 2, such as *CHT1* and *DLN142*, were strongly induced by H_2_O_2_ and to a lesser extent by Fe toxicity (Figures 4 and S2). The expression showed a clear dose dependency toward H_2_O_2_, with expression sharply increasing with 0.2, 1, and 5 mM of H_2_O_2_. The induction by Fe concentration was less pronounced than that of H_2_O_2_, and 20–80 mg L^−1^ Fe already saturated the expression. Consistent with the eigengene expression of group 15, all *STAR2, MGT1*, and *Os04g0450000* that encode a Zn‐finger protein were clearly induced by increasing Fe concentrations, while no clear trend was observed with H_2_O_2_ treatment (Figure 4). The eigengene analysis showed that genes in groups 3 and 10 were suppressed by both H_2_O_2_ and Fe toxicity, with a stronger effect of the former at an early time point. *UPS1* and *PRX11* in group 3, and *CYCB1*; 1 in group 10 indeed showed a strong response to H_2_O_2_, with 0.2 mM H_2_O_2_ already showing a significant suppressive effect. These genes were affected by Fe toxicity to a lesser extent.

### 3.5 Contribution of ART1 in Transcriptional Reprogramming Under Fe Toxicity in Rice Roots

**FIGURE 5:**
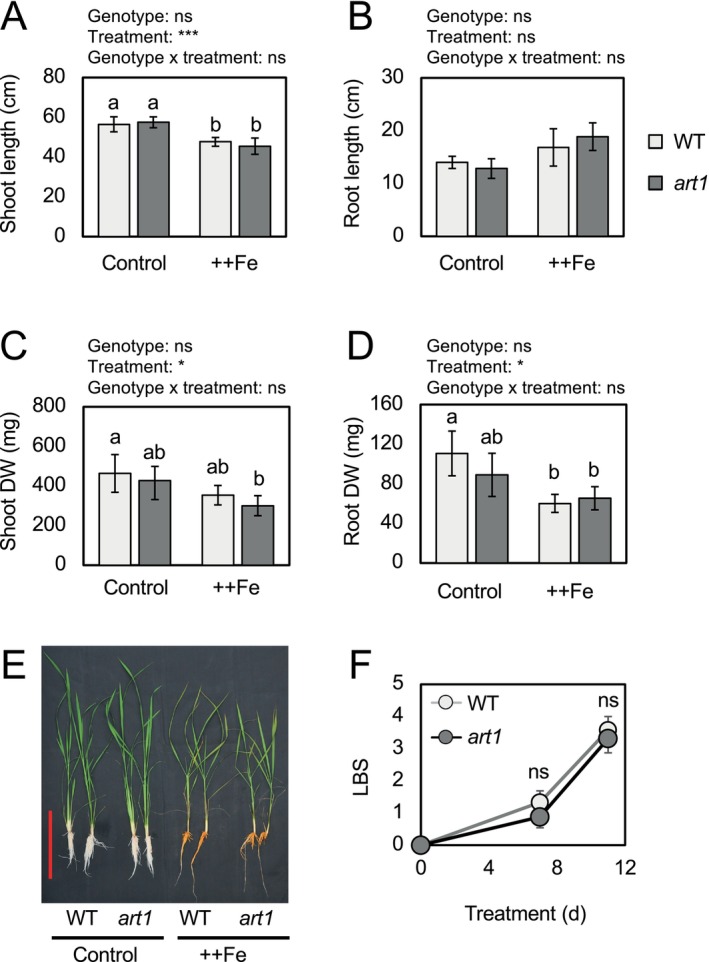
Phenotypic evaluation of WT and *art1* mutant plants under control and Fe toxicity conditions. (A, B) Length of shoot (A) and root (B). (C, D) Dry weight (DW) of shoot (C) and root (D). (E) Representative photo of plants under different treatments. The scale bar represents 20 cm. (F) Leaf bronzing score (LBS) under Fe toxicity conditions. Data represent mean ± standard deviation (*n* = 6–8). In A–D, two‐way ANOVA was conducted, and the significance for genotype, treatment, and genotype × treatment interaction is indicated with asterisks above each graph: Ns, *p* > 0.05; *p < 0.05; ***p < 0.001. Significant differences among sample groups are shown by different alphabets. In (F), a Student's *t*‐test was conducted at each time point, and ns indicates *p* > 0.05.

Co‐expression and meta‐analysis suggested the importance of ART1 in regulating genes under Fe toxicity in rice roots. To validate the contribution of ART1 in transcriptional regulation under Fe toxicity in rice roots, a previously characterized knock‐out mutant of ART1 (*art1*) (Huang et al. 2012) was grown alongside wild‐type Nipponbare (WT) plants with different nutrient solutions, and their growth phenotype was evaluated. The nutrient conditions included standard 1× Yoshida solution (2 mg L^−1^ Fe as Fe‐EDTA) and Fe toxicity solution (2 mg L^−1^ Fe as Fe‐EDTA +300 mg L^−1^ Fe as FeSO_4_), both of which contained 0.1% of melted agar (Materials and Methods). After 11 days of treatment, growth parameters, including shoot length, root length, shoot DW, and root DW, were not significantly different between WT and *art1* (Figure 5A–D). The extent of Fe toxicity‐induced leaf bronzing evaluated by leaf bronzing score (LBS) (Wu et al. 2014) was not significantly different between WT and *art1* at either 7 or 11 days after the onset of the treatment (Figure 5E,F).

**FIGURE 6:**
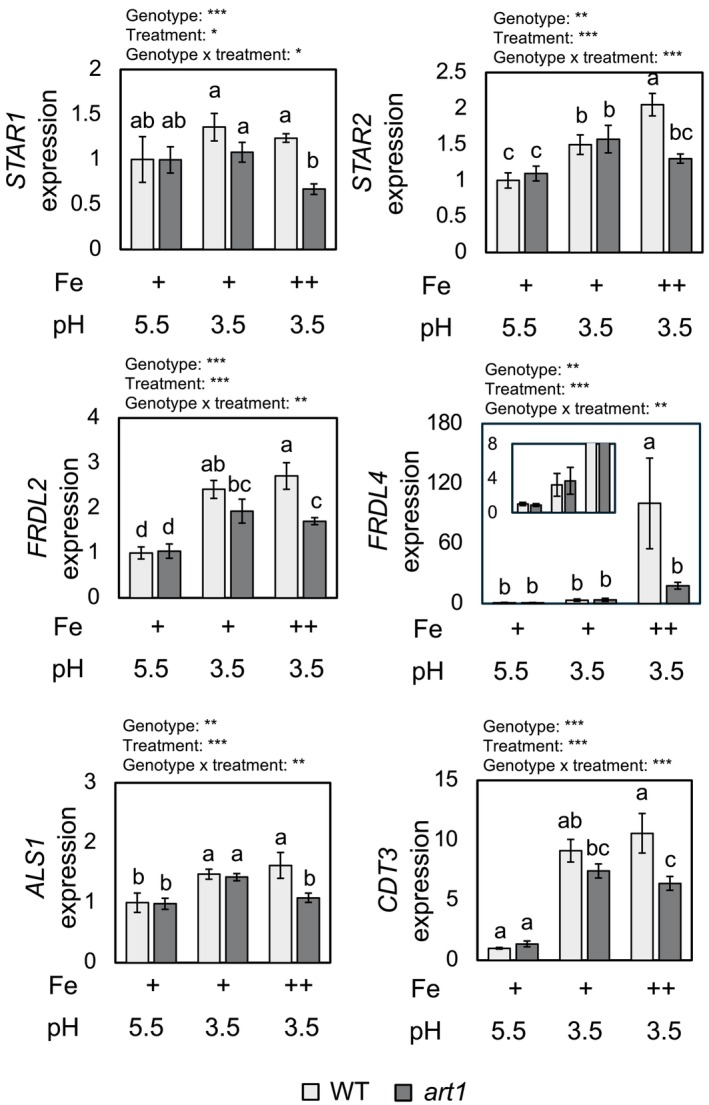
Expression of ART1‐target genes in WT and *art1* plants under control, low pH, and Fe toxicity conditions. The expression of ART1‐target genes in roots is shown. “+” and “++” denote standard (2 mg L^−1^ Fe‐EDTA) and excess (2 mg L^−1^ Fe‐EDTA + 300 mg L^−1^ FeSO_4_) Fe conditions. Two‐way ANOVA was conducted, and the significance for genotype, treatment, and genotype × treatment interaction is indicated with asterisks above each graph: *p < 0.05; **p < 0.01; ***p < 0.001. Significant differences among sample groups are shown by different alphabets. Data represent mean ± standard deviation (*n* = 4).

Gene expression in the whole root was examined after 3 h from the onset of the treatment. To eliminate biases caused by excess Fe, a treatment solution with low pH (3.5) and low P (5 μM) was also prepared. We note that such low pH levels are commonly observed in paddy fields affected by Fe toxicity (Sahrawat 2005). Significant interactions between treatment and genotype were observed for 6 of the examined genes, for which genotype and treatment effects were also significant (Figure 6). *STAR1* was not clearly induced by Fe toxicity, but *art1* decreased its expression under the Fe toxicity condition, leading to a significant difference with WT under this condition. The expression of *STAR2* was significantly induced by low pH, with Fe toxicity having an additional effect on WT. *art1* significantly induced the expression of *STAR2* to the same level as WT under the low pH condition but not under the Fe toxicity condition, contrary to WT (Figure 6). *FRDL2*, *ALS1*, and *CDT3* were induced by both low pH and Fe toxicity conditions. While *art1* showed a similar level of expression as WT under the low pH condition, it was significantly lower than WT under the Fe toxicity condition (Figure 6). The clearest contrast was observed in *FRDL4*, of which expression was very low under the control condition. While low pH had a small and non‐significant effect on its expression in both genotypes, its expression was highly induced in WT by Fe toxicity but not in *art1*. The attenuated response to Fe toxicity conditions in *art1* was confirmed in an independent experiment, with all genes having a significant difference between WT and *art1* only under Fe toxicity conditions or being induced by Fe toxicity only in WT (Figure S4). On the contrary, the expression of another putative ART1‐target gene, *MGT1*, did not clearly show genotype dependency, although it was significantly induced by Fe toxicity (Figure S5).

## 4 Discussion

### 4.1 Complexity of Transcriptional Regulation Under Fe Toxicity Stress in Rice Roots

Compared to other major nutrient stresses such as nitrogen or phosphorus deficiency (Gaudinier et al. 2018; Ueda et al. 2020; Prodhan et al. 2022), much less is known about the transcriptional regulatory mechanisms under Fe toxicity. The fact that ROS is generated under Fe toxicity (Wu et al. 2017; Takeuchi et al. 2024) and that both ROS‐ and nutrient‐related genes are involved in Fe toxicity tolerance (Aung, Kobayashi, et al. 2018; Aung et al. 2019; Li et al. 2019; Wu et al. 2019) indicates that both ROS and nutrient imbalances constitute the Fe toxicity response. However, this also contributes to the complexity of the Fe toxicity responses, which are dependent on tissues, Fe concentrations, and time points (Aung, Masuda, et al. 2018; Kakei et al. 2021). As a result of such complexity, no transcription factor involved in the Fe toxicity response in rice has been identified thus far. Thus, one objective of our study was to distinguish ROS‐dependent transcriptional regulations from the authentic nutrient‐related responses via a systems biology approach and seek a novel factor contributing to transcriptional regulation under Fe toxicity.

### 4.2 Changes in Gene Expression Induced by ROS and Nutrient Disorder

ROS‐responsive genes accounted for > 40% of the whole transcriptome, with their contributions being more prominent in the early phase of the treatment (3 h). The ratio gradually declined over time but still remained relatively high (> 25%) at 3 days (Figure 1E). Co‐expression analysis consistently showed significant contributions of ROS to the Fe toxicity response, with group 3 showing a common response pattern to Fe toxicity and H_2_O_2_ harboring many (2220) genes. These results indicate that the early response to Fe toxicity, especially for upregulated genes, is more represented by ROS stress, which may quickly trigger responses without involving alterations in nutrient status. This trend is reversed at later time points, with ROS‐unrelated, nutrient disorder‐related genes being more predominant over ROS‐related genes. Even though the amplitude of ROS response diminishes over time, the constitutive up‐ or down‐regulation of ROS‐responsive genes by excessive Fe aligns with continuous production of ROS under Fe toxicity conditions, indicating that the ability to cope with ROS is indeed an important tolerance mechanism for longer‐term Fe toxicity stress.

Much of the ROS sensing and responses are triggered via changes in the redox status of already existing molecules or protein phosphorylation, which do not require de novo synthesis of biomolecules (Huang et al. 2020; Mittler et al. 2022). Contrary to the ROS responses that occur relatively on a short time scale, many of the responses to nutrient disorders involve hormone synthesis or changes in cellular nutrient status, with the amplitude of the response being greater for increased duration of stress treatment (Misson et al. 2005). Thus, the larger contribution of ROS in the early phase of the Fe toxicity response could be due to the different speeds needed to activate ROS‐related and nutrient disorder‐related components.

Genes in group 15 (midnightblue) were minimally affected by ROS and were constitutively upregulated by Fe toxicity throughout the treatment period (Figure 2C; Table S2). Thus, genes in this group, such as *STAR2* and *Os04g0450000* encoding a Zn‐finger protein, may serve as authentic nutrient stress markers, contrary to ROS‐responsive genes such as *CHT1* and *DLN142* (Figure 4). The number of nutrient disorder‐responsive genes (i.e., genes other than ROS‐responsive genes) remained almost constant for upregulated genes over time but drastically increased for the downregulated genes at the late time point (Figure 1E). This represents the increased amplitude of nutrient disorder‐responsive genes, possibly linked with changes in cellular nutrient concentrations.

Typically, key factors captured by gene co‐expression analyses are limited to those that are transcriptionally regulated. This is because genes that are regulated by post‐translational modification without involving changes in mRNA expression are not classified into a meaningful gene group relevant to the stress of interest. In this study, we integrated previously identified nutrient‐responsive genes and identified ART1 as the novel transcription factor involved in the Fe toxicity response in rice roots. Since the expression of *ART1* is not strongly altered by either H_2_O_2_ or Fe toxicity stress in the root (Figure S6), co‐expression analysis alone would not have identified the involvement of ART1 in transcriptional regulation under Fe toxicity. Thus, such a combined strategy might be effective in gaining insight into important factors that are not transcriptionally regulated.

### 4.3 Functions of ART1 and Other Homologs Under Fe Toxicity

An Arabidopsis ( *Arabidopsis thaliana* ) C2H2 Zn‐finger transcription factor, SENSITIVE TO PROTON RHIZOTOXICITY (STOP1) (Iuchi et al. 2007), is considered a functional homolog of ART1 (Yamaji et al. 2009). STOP1 was initially identified as the master regulator for Al and low pH response in Arabidopsis (Iuchi et al. 2007), but a more recent study suggested its involvement in Fe toxicity response. The activity of STOP1 is primarily regulated at the post‐translational level by changing the localization between the cytosol and nucleus (Balzergue et al. 2017). Under P‐deficient and acidic conditions, Fe and Al induce nuclear accumulation of STOP1 with similar kinetics, leading to the activation of target genes such as *ALMT1*, which encodes a malate efflux transporter (Godon et al. 2019).

Despite the similar level of Al and Fe that induces STOP1 nuclear accumulation in Arabidopsis (Godon et al. 2019), the effects of ART1 varied greatly in Al and Fe toxicity responses in rice. In a previous study, *STAR2* was induced by > 5 folds by Al stress (μM order) in WT roots, with *art1* showing no induction (Yamaji et al. 2009). This contrasts with our current investigation, where we only observed ~2 fold induction by Fe stress (mM order) (Figure 5). Considering that *ART1* expression is not altered under Al or Fe toxicity stress (Yamaji et al. 2009) (Figure S6), ART1 is likely under post‐translational regulation, similar to STOP1, with a stronger effect caused by Al and a marginal effect by Fe toxicity. The different degrees of contribution of ART1 under Al and Fe stress in rice could be attributed to other post‐translational modifications, similar to Arabidopsis RAE1 and STAR1 that control the stability of STOP1 (Zhang et al. 2019; Fan et al. 2024). It is tempting to investigate the mechanism of ART1 regulation under Al and Fe toxicity conditions.

Despite a significant effect of ART1 on transcriptional regulation under Fe toxicity, *art1* plants did not exhibit altered tolerance levels, with no genotype effect or genotype and treatment interaction observed in any of the growth parameters (Figure 6). This contrasts with the large contribution of ART1 in Al tolerance reported previously, which mainly affected root growth and accounted for a significant genotypic difference in Al tolerance (Yamaji et al. 2009; Famoso et al. 2011; Arbelaez et al. 2017). In the case of Al stress, allelic variation causing amino acid polymorphism is suggested to underlie differences in the strengths of ART1 functions and plants' tolerance (Arbelaez et al. 2017). The cultivar Nipponbare used in the current study harbors the weaker allele of ART1, while IR64, a major indica variety, carries the stronger allele, which induces the expression of ART1‐target genes more strongly than the weaker allele (Arbelaez et al. 2017). Even though a significant effect of ART1 on Fe toxicity tolerance was absent in the Nipponbare genetic background, it may exert a greater influence on plants' performance under Fe toxicity in the IR64 background. It is also conceivable that ART1 homologs, some of which are transcriptionally regulated (Figure S6), have a greater influence on Fe toxicity response than ART1 and affect plant performance. The ART1‐mediated transcriptional changes may function in parallel with previously suggested other transcription factor proteins important for Fe toxicity response, such as those in NAC and bHLH families (Kakei et al. 2021; Rajonandraina, Ueda, et al. 2023), and account for genotypic differences in Fe toxicity tolerance reported previously (Rajonandraina, Rakotoson, et al. 2023). Alternatively, shoot‐related traits, rather than the responses in the roots, could be more important in determining plant‐level tolerance, as previously suggested (Wu et al. 2017), and root‐specific functions of ART1 may not have resulted in altered tolerance. Further investigations are necessary to elucidate key transcriptional factors for gene regulations and tolerance, as well as the regulatory mechanism of ART1.

### 4.4 Physiological Significance of ART1‐Mediated Fe Toxicity Responses

The partial redundancy of Al and Fe toxicity response in rice roots via ART1 may be related to soil characteristics that induce these stresses. Both Fe toxicity and Al stress tend to occur in acidic soils such as those in Sub‐Saharan Africa and South America (von Uexküll and Mutert 1995; Becker and Asch 2005; Kochian et al. 2015). These metals strongly adsorb to phosphate and make it unavailable to plants (Hsu 1964; Gerke 2010), causing P deficiency. A previous study in Arabidopsis showed that the nuclear accumulation of STOP1 in response to Fe and Al occurs only under P‐deficient conditions and thus suggested that Fe and Al may bind to a common molecule in the cytosol to induce the responses (Godon et al. 2019). Although the physicochemical properties of soil that induce Fe toxicity and Al stress are different, sharing common mechanisms to respond to Fe toxicity and Al stress may have provided advantages to plants under certain soil types (e.g., acidic soils strongly fixing P) in their natural habitat. For example, this could be considered a mechanism that plants have developed to cope with P deficiency, which frequently coincides with Al and Fe stresses. A recent study showed that a malate efflux transporter from the roots of *Hakea laurina*, which is activated by Al stress, could be key for its high P acquisition efficiency (Yamada et al. 2025). Thus, the responses triggered by Arabidopsis STOP1 and rice ART1 could result in adaptive traits relevant to multiple stresses prevalent in acidic soil conditions. It would be intriguing to investigate if other components of Al responses, such as sensing of Al by a recently identified protein kinase ALR1 (Ding et al. 2024), are shared by Fe toxicity responses.

## 5 Conclusions

In conclusion, we propose that the complexity of transcriptional regulation under Fe toxicity is explained by temporal changes in the effects of ROS and nutrient disorders under Fe toxicity in rice roots: In the initial phase, when the plant's nutrient status has not been significantly affected, the effect of ROS is predominant. However, after a prolonged stress duration, nutrient imbalances would increase and their negative effects on transcriptional regulation may surpass the impact of ROS. By dissecting different components of the response, we revealed the contribution of a C2H2 Zn‐finger transcription factor, ART1, in shaping the transcriptional landscape under Fe toxicity in rice roots. This study provides clues on mechanisms of Fe toxicity response and will be valuable for manipulating the response toward Fe toxicity and tolerance.

## Author Contributions

Y.U. conceived the study, performed all the experiments, wrote the manuscript drafts, and prepared all figures and tables. N.Y. provided the *art1* mutant seeds. N.Y. and M.W. provided editorial comments. All authors have checked the final version of the manuscript and agreed with the submission.

## Disclosure

The authors have nothing to report.

## Supporting information


**Table S1.** Primers used in this study.
**Table S2.** Summary of RNA‐seq and co‐expression analysis.
**Table S3.** Result of gene ontology enrichment analysis.
**Table S4.** Summary of transcriptome data used for the meta‐analysis.

**Figure S1.** Concentration of Al in Fe solution measured by ICP‐AES.
**Figure S2.** Expression of eigengenes.
**Figure S3.** Concentration of P in the control and Fe toxicity solution.
**Figure S4.** Expression of ART1‐target genes in WT and *art1* plants in an independent experiment.
**Figure S5.** Expression of *MGT1* under Fe toxicity in roots.
**Figure S6.** Expression of *ART1* and its homologs under H_2_O_2_ and Fe toxicity stress in roots.

## Data Availability

The raw RNA sequence data have been submitted to the NCBI SRA database under accession number PRJNA1237919.
