# Supplementary material for: Comparative Transcriptome Reveals ART1‐Dependent Regulatory Pathways for Fe Toxicity Response in Rice Roots
Source: Physiol Plant. 2025 Jul 14;177(4):e70398. doi: 10.1111/ppl.70398 (PMC12257064; doi:10.1111/ppl.70398)
Supplement: Supplementary file 2 — Figure S1. Concentration of Al in Fe solution measured by ICP‐AES. Figure S2. Expression of eigengenes. Figure S3. Concentration of P in the control and Fe toxicity solution. Figure S4. Expression of ART1‐target genes in WT and art1 plants in an independent experiment. Figure S5. Expression of MGT1 under Fe toxicity in roots. Figure S6. Expression of ART1 and its homologs under H2O2 and Fe toxicity stress in roots. [file PPL-177-e70398-s002.pdf]

Comparative transcriptome reveals ART1-dependent regulatory pathways for Fe toxicity response in rice roots

Yoshiaki Ueda, Naoki Yamaji, Matthias Wissuwa

*Correspondence
Yoshiaki Ueda
E-mail: ueday0428@jircas.go.jp

Supplementary Files

Fig. S1 | Concentration of Al in Fe solution measured by ICP-AES
Fig. S2 | Expression of eigengenes
Fig. S3 | Concentration of P in the control and Fe toxicity solution
Fig. S4 | Expression of ART1-target genes in WT and *art1* plants an independent experiment
Fig. S5 | Expression of *MGT1* under Fe toxicity in roots
Fig. S6 | Expression of *ART1* and its homologues under H_2O_2 and Fe toxicity stress in roots

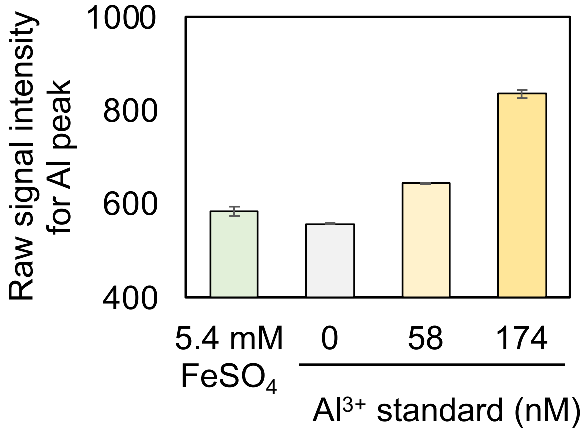

Fig. S1 | Concentration of Al in Fe solution measured by ICP-AES

The signal intensity of the peak representing Al^{3+} was measured for Al^{3+} standard and 5.4 mM $FeSO_4$, which is equivalent to the Fe concentration in the hydroponic solution for Fe toxicity treatment. Data represent mean ± standard deviation (n=3).

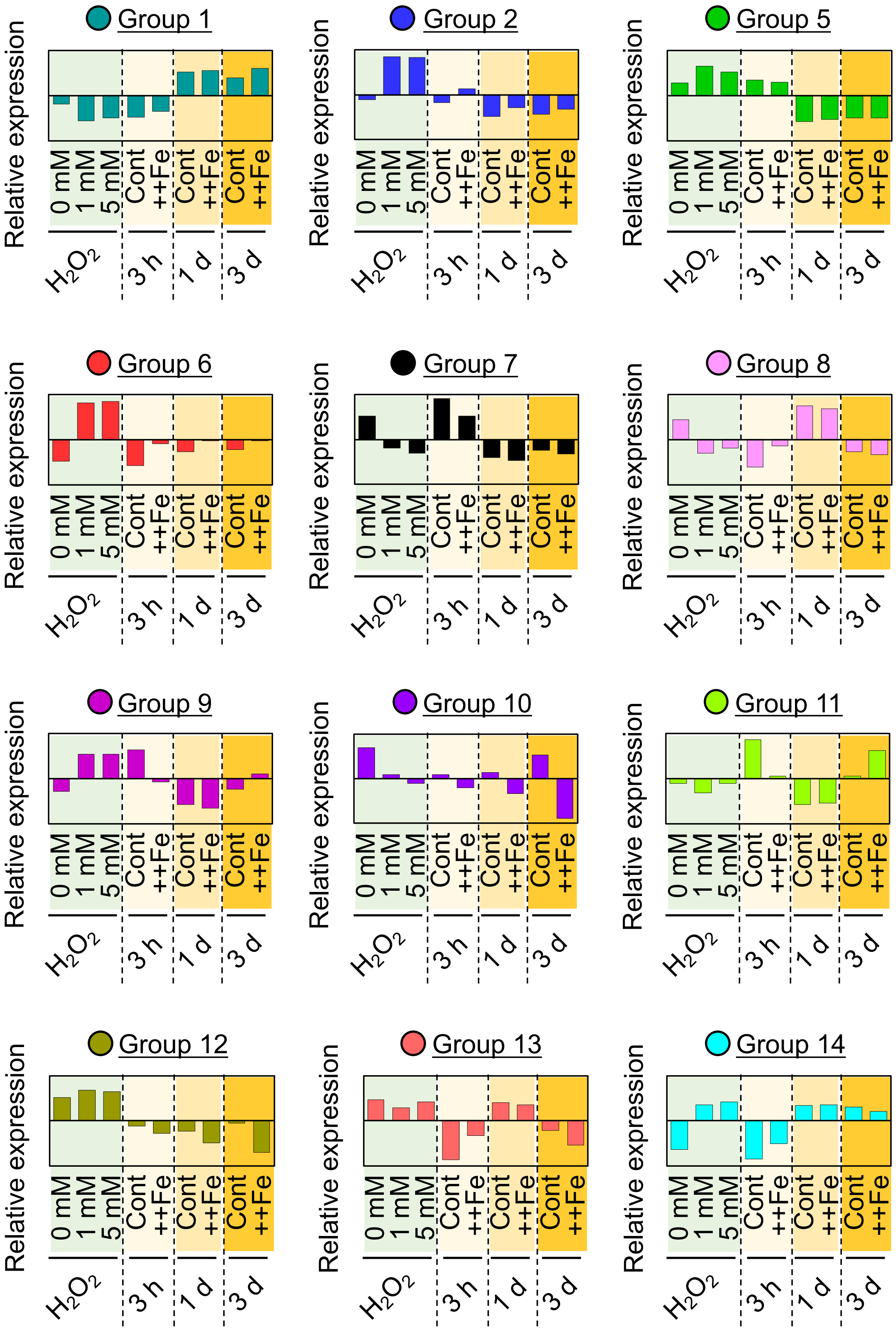
Group 1
Group 2
Group 5
Group 6
Group 7
Group 8
Group 9
Group 10
Group 11
Group 12
Group 13
Group 14
Relative expression
0 mM
1 mM
5 mM
Cont
++Fe
H_2O_2
3 h
1 d
3 d

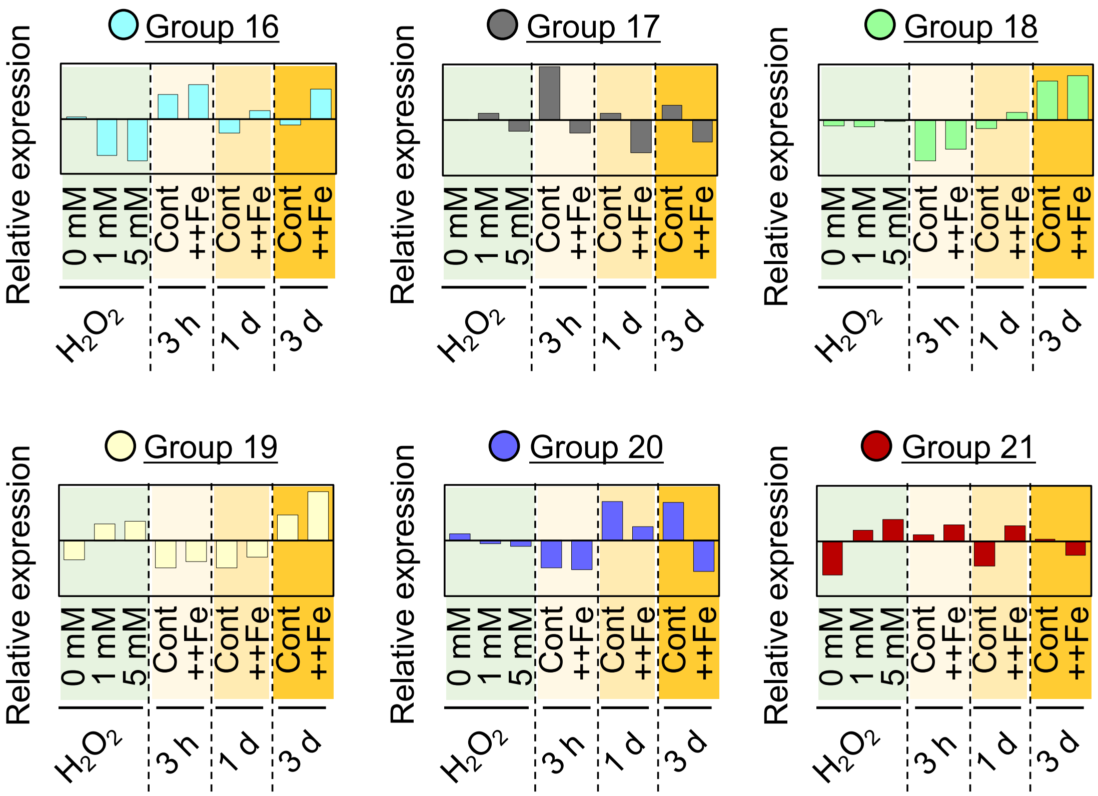

Fig. S2 | Expression of eigengenes

Eigengene expression of each sample was calculated for each condition and average of three plants are shown. The y-axis scale is consistent across all graphs.

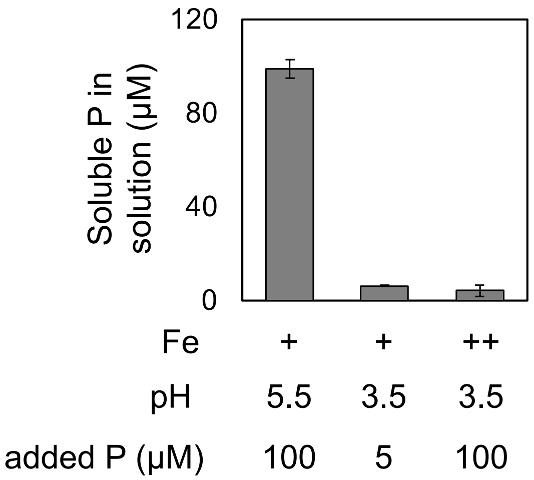

Fig. S3 | Concentration of P in the control and Fe toxicity solution

P concentration in the solution was measured by molybdenum blue method after removal of any precipitation. Data represent mean ± standard deviation (n=2). The data were obtained in Experiment 3.

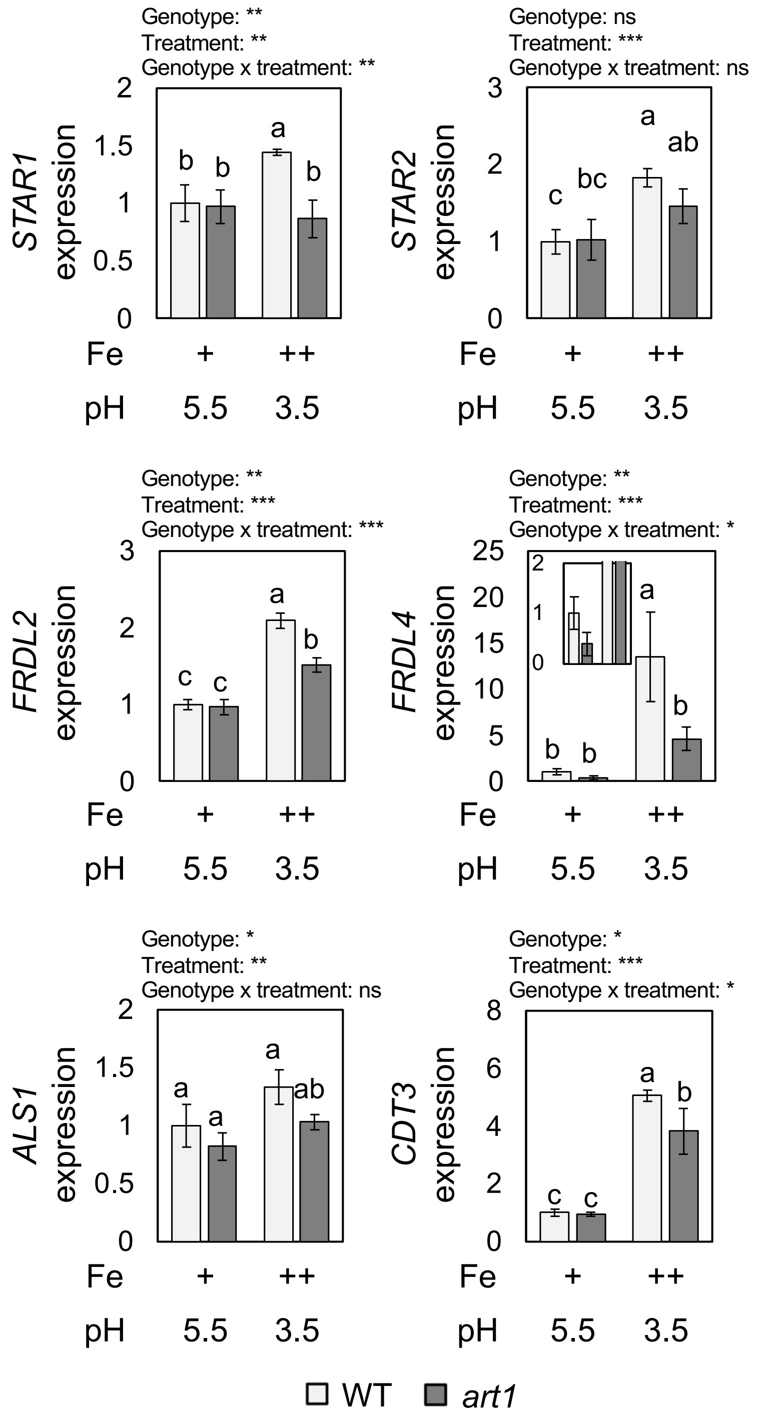

Fig. S4 | Expression of ART1-target genes in WT and *art1* plants an independent experiment

Data represent mean ± standard deviation (n=3-4). The data were obtained in Experiment 4.

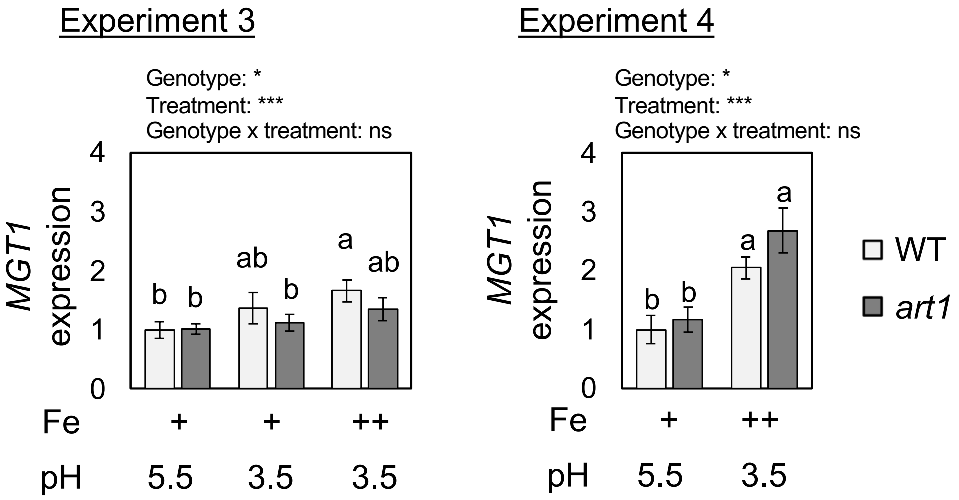

Fig. S5 | Expression of *MGT1* under Fe toxicity in roots

Data represent mean ± standard deviation (n=4 for Experiment 3, n=3-7 for Experiment 4).

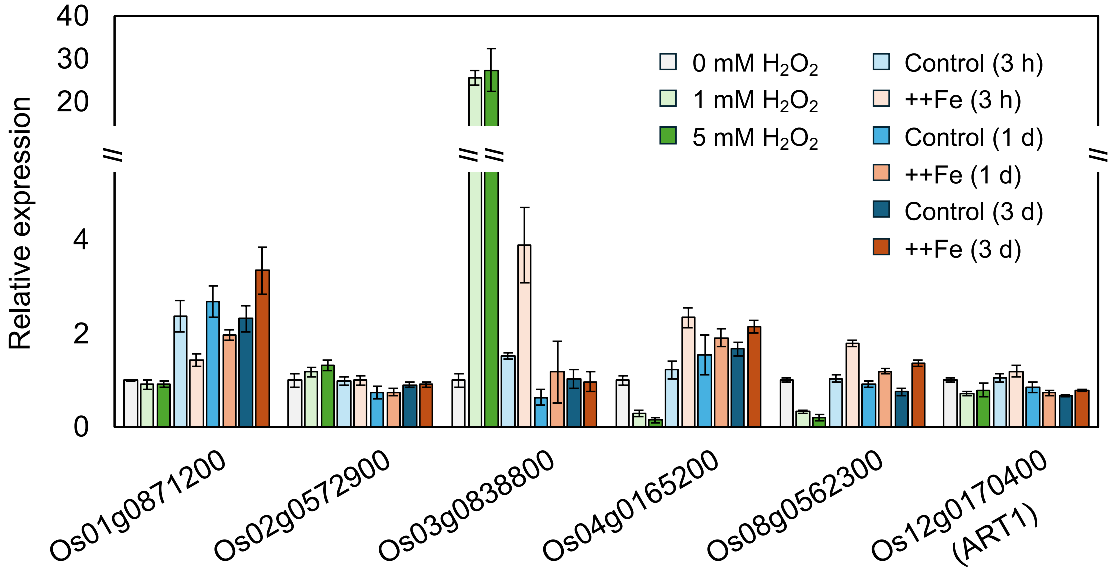

Fig. S6 | Expression of *ART1* and its homologues under H_2O_2 and Fe toxicity stress in roots

Expression obtained from RNA-seq under 0 mM H_2O_2 condition was defined as 1 and the other expression data are expressed as relative value. Data represent mean ± standard deviation (n=3).
